# Quality Improvement: The Power of Behavioural Change in Involvement

**DOI:** 10.1192/bjo.2024.418

**Published:** 2024-08-01

**Authors:** Geetika Singh, Mehtab Ghazi Rahman, Isaac Obeng, Lucy Palmer, Janet Seale

**Affiliations:** Central and North West London NHS Foundation Trust, London, United Kingdom

## Abstract

**Aims:**

Aim:

80% of Central and North West London NHS Foundation Trust (CNWL) QI projects will have meaningful Service User & Carer involvement by August 2023 (baseline was 46%).

Background:

Service user and carer (SU&C) involvement is increasingly recognised as integral to healthcare improvement efforts. However, despite its many benefits, the meaningful involvement of service users and carers remains a challenge.

Thus, it is necessary to get an in-depth understanding of the barriers and enablers to embedding involvement in improvement practice at the individual, service and organisational levels. With this understanding and staff can then co-produce evidence-informed behaviour change interventions to improve SU&C involvement.

Patient representation and lived experience

This improvement work embraces a full and continuous partnership with SU&Cs.

SU&C worked with CNWL staff in the conception, execution, delivery and dissemination of this work including EbE Improvement Forum.

They, thus, serve as integral members of the project team where they provide valuable input based on their lived experiences and perspectives to help shape the direction of the work. As equal partners, it also helps foster a culture of mutual respect, collaboration, and trust between all the parties involved.

**Methods:**

This work adopts the COM-B model. The approach to conduct semi-structured interviews (interview questions were based on COM-B model and behavioural change wheel) with frontline healthcare staff and SU&Cs. The interviews gave insights on the barriers and facilitators to SU&C involvement in healthcare improvement work.

This then generated operational-level and actionable change ideas to guide tailored strategies for enhancing involvement capabilities, widening involvement opportunities and enabling motivations using the model for Improvement. These change ideas were then co-tested with SU&C using the Model for Improvement approach.

This systematic approach enabled a cultural shift towards collaborative partnerships between healthcare staff and SU&C to contribute to the service improvement.

Measurement of improvement

1. Qualitative data to understand enablers and barriers to involvement in improvement work.
2. Percentage of all QI projects (registered on Life QI and scoring 1 or more on IHI Project Score) that have a SU&C involvement.

**Results:**

Effects of changes

Staff and SU&C interviews identified the key barriers as inadequate capability (lack of understanding and skills), limited opportunities (leadership, resources, access) and insufficient motivation (discomfort, inability, time limitations). Enablers included appreciating diverse perspectives, leadership support and buy-in, established processes, valuing insights and patient empowerment.

The outcome measure also showed an increase in the number of improvement projects at CNWL that have SU&C involvement from 46% to 80%.

**Conclusion:**

Lesson Learnt:

This work has shown that by bringing multi-disciplinary staff and SU&Cs together generates cognitive diversity to the learning to drive improvement and sustain the gains. Furthermore, partnership working helped to create and establish learning culture within the healthcare service.

Message to others:

1. Strong executive sponsorship helps to drive involvement across the organisation.
2. By bringing staff and SU&Cs together generates cognitive diversity to the learning to drive improvement.
3. Using well-known and established behavioural change model, such as COM-B model, helped to identify, design and synthesize behaviour change interventions.

